# Ganglioside GM3 Synthase Deficiency in Mouse Models and Human Patients

**DOI:** 10.3390/ijms23105368

**Published:** 2022-05-11

**Authors:** Kei-ichiro Inamori, Jin-ichi Inokuchi

**Affiliations:** 1 Division of Glycopathology, Institute of Molecular Biomembrane and Glycobiology, Tohoku Medical and Pharmaceutical University, Sendai 981-8558, Miyagi, Japan; 2 Forefront Research Center, Graduate School of Science, Osaka University, Toyonaka 560-0043, Osaka, Japan

**Keywords:** gangliosides, GM3 synthase, *ST3GAL5*, glycosyltransferase, GM3S deficiency

## Abstract

Gangliosides (glycosphingolipids containing one or more sialic acids) are highly expressed in neural tissues in vertebrates, and four species (GM1a, GD1a, GD1b, GT1b) are predominant in mammalian brains. GM3 is the precursor of each of these four species and is the major ganglioside in many nonneural tissues. GM3 synthase (GM3S), encoded by *ST3GAL5* gene in humans, is a sialyltransferase responsible for synthesis of GM3 from its precursor, lactosylceramide. *ST3GAL5* mutations cause an autosomal recessive form of severe infantile-onset neurological disease characterized by progressive microcephaly, intellectual disability, dyskinetic movements, blindness, deafness, intractable seizures, and pigment changes. Some of these clinical features are consistently present in patients with *ST3GAL5* mutations, whereas others have variable expression. GM3S knockout (KO) mice have deafness and enhanced insulin sensitivity, but otherwise do not display the above-described neurological defects reported in *ST3GAL5* patients. The authors present an overview of physiological functions and pathological aspects of gangliosides based on findings from studies of GM3S KO mice and discuss differential phenotypes of GM3S KO mice versus human GM3S-deficiency patients.

## 1. Introduction

**Figure 1:**
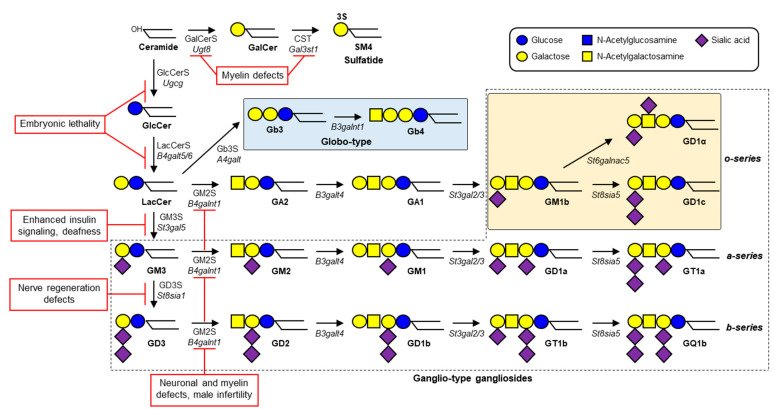
Biosynthetic pathway of gangliosides and sulfatide. GlcCerS catalyzes with the addition of glucose to ceramide, and LacCerS subsequently adds galactose to GlcCer to form LacCer. GalCerS catalyzes with the addition of galactose to ceramide to form GalCer, and CST subsequently adds a sulfate group to form sulfatide SM4. Red rectangles indicate phenotypes observed in mouse KO models. Expression of globo-type GSLs (highlighted by blue rectangle) is elevated substantially in fibroblasts of human GM3S-deficiency patients. In contrast, in GM3S KO-mouse brains and fibroblasts, expression of globo-type GSLs is only slightly elevated, but that of o-series gangliosides (GM1b, GD1α, GD1c: highlighted by yellow rectangle) is elevated substantially (see main text).

Gangliosides—glycosphingolipids (GSLs) that contain one or more sialic acids—are fundamental components of cell membrane microdomains involved in dynamic regulation of various cell physiological processes [1]. Ganglioside biosynthesis is initiated by the addition of a glucose residue to ceramide to form glucosylceramide (GlcCer); this process is catalyzed by GlcCer synthase (GlcCerS, encoded by *Ugcg* gene in mice) (Figure 1). Similarly, a galactose residue can be added to ceramide to form galactosylceramide (GalCer), catalyzed by GalCer synthase (GalCerS, encoded by *Ugt8* gene). Synthesis of GlcCer occurs on the cytosolic surface of Golgi, whereas GalCer synthesis occurs on the luminal surface of endoplasmic reticulum [2,3]. Sulfation of GalCer by cerebroside sulfotransferase (CST, encoded by *Gal3st1* gene) generates sulfatide SM4, a major lipid component of myelin sheath in both the central nervous system (CNS) and peripheral nervous system (PNS). GlcCer is galactosylated by lactosylceramide (LacCer) synthase (LacCerS, encoded by *B4galt5* or *B4galt6*) to form LacCer, the precursor of a variety of ganglioside species and other types of GSLs. GM3 synthase (GM3S, encoded by *St3gal5* gene) is a sialyltransferase that adds a sialic acid residue to LacCer to initiate synthesis of a- and b-series gangliosides. GD3 synthase (GD3S, encoded by *St8sia1* gene) is another sialyltransferase that catalyzes formation of disialoganglioside GD3. GM2 synthase (GM2S, encoded by *B4galnt1* gene) adds N-acetylgalactosamine (GalNAc) to LacCer, GM3, or GD3 to generate (respectively) GA2, GM2, or GD2. Gangliosides are expressed in essentially all vertebrate tissues and cells, most abundantly in the nervous system. GM1, GD1a, GD1b, and GT1b in particular are the predominant ganglioside species in mammalian brain tissues [4].

Gangliosides interact with various transmembrane receptors and regulate their functions in signal transduction pathways; such receptors include the epidermal growth factor receptor (EGFR), platelet-derived growth factor receptor (PDGFR), the vascular endothelial growth factor receptor (VEGFR), the hepatocyte growth factor receptor (c-Met), the nerve growth factor receptor (TrkA), the insulin receptor (InsR), the insulin-like growth factor 1 receptor (IGF1R), and the leptin receptor [5,6,7,8]. GM3 regulates EGFR and InsR signaling through lateral interactions with these receptors, and development of insulin resistance involves increased GM3 expression during obesity-induced inflammation [9,10,11,12]. The authors demonstrated recently that GM3 species with varying acyl-chain lengths or degrees of α-hydroxylation or desaturation are secreted abundantly into human serum, and modulate Toll-like receptor 4 (TLR4) activation in an acyl-chain structure-dependent manner [13].

## 2. Neuronal Phenotypes of Ganglioside-Deficient Mouse Models

Mouse models with GlcCerS (*Ugcg*) deletion lack downstream GSLs, including LacCer, GM3, and derived gangliosides, and are embryonic lethal [14] (Figure 1). Nestin–Cre transgenic mice with neuronal *Ugcg* deletion displayed severe ataxia and cerebellar and peripheral nerve dysfunction shortly after birth and died within 24 days [15]. A study of L7-Cre transgenic mice in which Cre-dependent *Ugcg* deletion occurs specifically in Purkinje cells by week (wk) 3 after birth showed that axonal GlcCer-derived GSLs are essential for axonal homeostasis and normal myelin sheath formation [16]. In contrast, no myelin abnormalities resulted from oligodendrocyte-specific *Ugcg* deletion using Cnp–Cre [17]. These and other studies demonstrate the essential roles of axonal GSLs, particularly gangliosides, in neuronal function and axon-myelin interactions.

LacCerS is encoded by either one of two genes, *B4galt5* and *B4galt6*. *B4galt5* knockout (KO) mice were embryonic lethal because of extra-embryonic defects, whereas growth was normal for *B4galt6* KO mice [18,19,20]. Growth also appeared normal for mice with neuronal *B4galt5* deletion using Nestin–Cre (*B4galt5* cKO) and also showed apparently normal growth. Double KO (DKO) mice for *B4galt5* and *B4galt6* (*B4galt5*/*B4galt6* DKO) were generated by crossing *B4galt5* cKO and *B4galt6* KO mice to completely eliminate LacCerS from the CNS [21]. These mice appeared normal at birth but displayed growth retardation and motor deficits by wk 2 and died by wk 4, similarly to neuronal *Ugcg* KO mice. LacCerS activity level was zero in DKO brain, and around half of normal control value in *B4galt5* cKO and *B4galt6* KO brains, indicating that both *B4galt5* and *B4galt6* genes are necessary for normal LacCerS activity in CNS. DKO mice had impaired neurite outgrowth and perineuronal net formation, presumably because of the absence of GM1 and other gangliosides. Laminin/GM1 interaction is important for nerve growth factor signaling to induce clustering of GM1, TrkA, and β1 integrin [22].

DKO mice also showed severe impairment of axon and myelin formation, presumably as a result of the lack of interaction between ganglioside and myelin-associated glycoprotein (MAG). GM3S (*St3gal5*) KO mice, in contrast to neuronal *Ugcg* KO and *B4galt5*/*B4galt6* DKO mice, showed no apparent neuronal abnormalities [23]. One possible explanation is that o-series gangliosides have an alternative synthetic pathway not dependent on GM3S (Figure 1). MAG (Siglec-4), a member of the Siglec (sialic acid-binding immunoglobulin-type lectin) family, is expressed on the innermost myelin layer in oligodendrocytes and Schwann cells, and functions as a binding partner of axonal gangliosides (GD1a, GT1b) based on recognition of their Neu5Acα2-3Galβ1-3GalNAc sequence [24]. GM2S (*B4galnt1*) KO mice, which lack a MAG-binding sequence, display reduced myelination and progressive axonal degeneration in both CNS and PNS as they age and a phenotype similar to that of MAG KO mice [25]. The phenotype of GM2S/MAG DKO mice was similar to those of GM2S KO and MAG KO, indicating that ganglioside/MAG interaction is essential for myelin and axonal integrity. GM3S KO mice lack GD1a and GT1b; however, their brain has a high content of unusual o-series gangliosides GM1b and GD1α, which contain a terminal MAG-binding sequence. This finding suggests that the alternative expression of o-series gangliosides compensates for lack of GD1a and GT1b.

GM3S/GM2S (*St3gal5*/*B4galnt1*) DKO mice, which lack all ganglio-type gangliosides, have small brains, develop severe neurodegenerative disease with axonal regeneration and disrupted myelin interaction, and die around an age of 4–5 months [26]. GM2S KO mice also lack GM1 and a MAG-binding sequence, but their neuronal phenotype is less severe than that of DKO, suggesting that GM3 and/or other gangliosides compensate for the lack of GM1 and laminin interaction. The phenotype of GM3S/GM2S DKO is less severe than that of neuronal *Ugcg* KO or *B4galt5*/*B4galt6* DKO, suggesting that other LacCer-derived GSLs also play essential roles in axon/myelin interaction and/or other neuronal functions.

GD3S (*St8sia1*) KO mice, which lack b-series gangliosides, display mostly normal development with only subtle deficiencies, i.e., reduced regeneration of axotomized hypoglossal nerves, thermal hyperalgesia, mechanical allodynia, and reduced response to prolonged noxious stimuli [27,28,29]. GD3S/GM2S (*St8sia1*/*B4galnt1*) DKO mice, which express GM3 as major ganglioside, were generated independently by two groups in 2001–2002. R.L. Proia’s group reported that these mice appear normal at birth, but soon display neurodegeneration and sudden lethal audiogenic seizures [29]. K. Furukawa’s group, using a similar DKO model, observed that mice do not have seizures, but display reduced sensory nerve sensitivity resulting from nerve degeneration, and consequent over-scratching and skin lesions [30]. In a follow-up 2009 study, they observed that these mice have dysregulated complement activation, and consequent inflammation and neurodegeneration [31].

## 3. Phenotype of GM3S KO Mice

### 3.1. Metabolic Phenotype

GM3S (*St3gal5*) KO mice are born with typical Mendelian genotypic ratios and display no major abnormalities, presumably because of alternative expression in brain of o-series gangliosides, as described above. Proia’s group reported that these mice display enhanced phosphorylation of InsR in skeletal muscle following insulin injection, enhanced glucose and insulin tolerance, and reduced susceptibility to high-fat diet (HFD)-induced insulin resistance [23]. Chronic inflammation in adipose tissue evidently contributes to development of systemic insulin resistance [32]. Our group observed elevated GM3 expression in adipose tissues of obese model Zucker *fa/fa* rats and *ob/ob* mice, and of HFD-induced obese wild-type (WT) C57BL/6 mice, which have upregulated TNF-α expression in adipose tissues and develop insulin resistance [12,33]. GM3S KO mice, under HFD conditions, had body weight gain similar to that of WT; however, they had significantly lower TNF-α level in adipose tissue and higher gene expression of anti-inflammatory cytokine IL-10 and of adiponectin, indicating that they were resistant to the development of a chronic, low-grade inflammatory state in visceral fat tissues under HFD condition [12]. Their glucose tolerance test (GTT) and insulin-tolerance test (ITT) results under HFD condition were better than those of WT.

GM3S KO mice of KK-*Ay* genetic background (unlike those of C57BL/6 background) had significant differences in obese phenotype in comparison with WT KK-*Ay* mice, which displayed early, severe onset of obesity and diabetic pathology [8]. WT KK-*Ay* were hyperphagic and developed morbid obesity with associated glucose intolerance and insulin resistance. In contrast, KK-*Ay*/GM3S KO had a significantly lower food intake and body weight, and better glucose and insulin tolerance. Hypothalamic sensitivity to i.p.-administered leptin at age 10 wks in WT KK-*Ay* was largely gone (because of development of “leptin resistance”) but was still present in KK-*Ay*/GM3S KO. GM3S KO cells generated from hypothalamic neuronal cell line N-41 did not express a-series gangliosides but did express o-series ganglioside GM1b (usually a minor component), similarly to GM3S KO brain. GM3S KO cells had reduced leptin-dependent STAT3 phosphorylation, which is required for leptin anti-obesity activity [34,35], consistently with observations in other KO models that a-series gangliosides positively regulate LepR signaling [6,7]. In contrast, leptin-dependent ERK phosphorylation was strongly enhanced in GM3S KO cells. Leptin-dependent ERK phosphorylation in hypothalamus, mediated by its positive regulator SH2-containing phosphatase-2 (SHP-2), plays an important role in energy balance regulation [36,37,38]. Our findings therefore suggest that LepR-ERK pathway is enhanced in GM3S KO mice and opposes leptin resistance. Coat color is yellowish in WT KK-*Ay* mice because of disrupted melanocortin signaling, but grayish in KK-*Ay*/GM3S KO mice, suggesting that GM3 is involved in melanin synthesis, presumably through melanocortin 1 receptor (MC1R) signaling. GM3 or related gangliosides thus appear to play important roles in leptin and melanocortin signaling. Detailed mechanisms for such activity remain to be investigated.

### 3.2. Deafness

Mice normally begin to recognize specific sounds by postnatal day 12 (P12). In WT cochlea, GM3 is the major GSL on P1, and subsequent to P3 there is a progressive increase in GM3 and in other GSLs, including GlcCer, complex gangliosides (GM1, GD1a, GD3, GD1b, GT1b), and sulfatides [39]. GM3S KO mice, whose cochlea lack a- and b-series gangliosides and contain o-series gangliosides GM1b and GD1α instead, display impaired hearing ability initially, and lose the ability completely after a few days. In contrast, GM2S KO mice, whose cochlea contain only GM3 and GD3, have apparently normal hearing ability [39,40]. In GM3S KO mice, electrophysiological and histological auditory system analyses revealed selective degeneration of the organ of Corti in the cochlea. In WT mice, GM3 and GM1 are expressed in different manners in outer hair cells (OHCs) and inner hair cells (IHCs), two types of sensory cells in the organ of Corti. GM3 is expressed in both IHCs and OHCs, whereas GM1 is expressed only in IHCs. Transmission electron microscopic examination of GM3S KO OHCs revealed numerous membrane blebs and intracellular vesicles composed of membranous structures, indicating impaired vesicular trafficking. Stereocilia of GM3S KO IHCs were fused, and protein tyrosine phosphatase receptor Q (PTPRQ), which is essential for stereocilia integrity, was maldistributed [39]. PTPRQ and myosin VI, an actin-based motor protein, jointly form a functional complex that mediates plasma membrane/cytoskeletal interaction [41]. Mice deficient in either PTPRQ or myosin VI are deaf and display a lack of tapered base and fusion of stereocilia [42,43]. These findings, taken together, suggest that (i) GM3-enriched membrane microdomains are necessary for formation and proper localization of PTPRQ/myosin VI complexes in cochlear hair cells; (ii) absence of GM3 results in disruption of PTPRQ/myosin VI complexes and consequent impairment of stereocilia integrity and auditory signal transmission capability.

### 3.3. Suppressed Activation of Helper T Cells

T cells interact with antigen-presenting cells at contact sites, termed immunological synapses, in which the T cell receptor (TCR)/major histocompatibility complex (MHC) aggregate. Formation of such synapses promotes signaling and subsequent T-cell activation, and lipid rafts are involved in this process [44]. Structural analysis of gangliosides in immature thymocytes from mouse lymphoid organs revealed that CD4+ and CD8+ T cells express six species (GM1a, GM1b, GD1b, GD1c, GalNAcGM1b, extended-GM1b) at differing levels [45]. In WT mice, GM1a levels are higher in CD4+ than in CD8+ T cells. In GM3S KO mice, CD4+ T cells have severe impairment of TCR-mediated proliferation and cytokine production, whereas CD8+ T cells function normally. Incubation of CD4+ T cells in these mice with GM3 or GM1a corrects the impairment. CD8+ T cells from WT mice express mainly o-series GA1, GM1b, GalNAcGM1b, and extended-GM1b. CD8+ T cells from GM2S KO mice have severe impairment of TCR-mediated proliferation and cytokine production, which is corrected by incubation with o-series GA1 and GM1b. GM3S KO mice do not develop experimental asthma, consistently with the above findings [45].

Allergic airway inflammation is mediated mainly by CD4+ Th2 cells that secrete effector cytokines (IL-4, Il-5, IL-13), which are responsible for asthma pathological processes, such as airway hyperresponsiveness and persistent eosinophilic inflammation [46]. In an experimental asthma model, ovalbumin (OVA)-sensitized mice were challenged by OVA inhalation. This treatment induced extensive mucus hypersecretion for WT but not for GM3S KO mice. Relative to WT, GM3S KO mice had significantly lower numbers of infiltrating cells (eosinophils, lymphocytes) in airways, and lower Th2 cytokine levels in bronchoalveolar lavage fluids. In adoptive transfer experiments, OVA-induced infiltration of inflammatory cells (eosinophils, lymphocytes) was suppressed in OVA-sensitized WT mice that received CD4+ T cells from OVA-sensitized GM3S KO mice. These findings indicate that CD4+ T cell activation depends specifically on a-series gangliosides, whereas CD8+ T cell activation depends on o-series gangliosides, presumably with a T-cell subset-specific raft microenvironment serving as a platform for TCR activation. Ongoing studies are focused on functions of specific ganglioside species for TCR activation in membrane microenvironments.

### 3.4. Impaired Cholesterol Uptake

Intestinal cholesterol absorption, which is necessary for maintenance of cholesterol homeostasis, is mediated by the transmembrane protein Niemann-Pick C1-like 1 (NPC1L1) [47], which acts as a cholesterol transporter. NPC1L1 has high levels on the apical side of enterocytes in the jejunum [48]. Lipid raft proteins flotillin-1 and -2 are combined with NPC1L1 to form cholesterol-enriched membrane microdomains. In mouse studies, flotillins were shown to play essential roles in NPC1L1-mediated cholesterol uptake, biliary cholesterol reabsorption, and homeostasis of plasma lipid levels [49]. In HEK293T (a highly transfectable derivative human cell line expressing a mutant version of SV40 large T antigen), GM3S KO resulted in the strong inhibition of NPC1L1-dependent cholesterol uptake and impairment of cholesterol-dependent NPC1L1 internalization, relative to cells [50]. GM3S KO mice were resistant to high-cholesterol-diet-induced hypercholesterolemia. In spontaneously hyperlipidemic ApoE-deficient (*ApoE^shl^*) mice, GM3S KO resulted in a significant reduction of plasma cholesterol levels, and of intestinal cholesterol absorption rate, assessed based on fecal analysis of mice orally gavaged with [^14^C] cholesterol. In small intestines of both *ApoE^shl^* and *ApoE^shl^* GM3S KO, in the absence of cholesterol feeding, NPC1L1 was localized mainly on the apical side of enterocytes. When cholesterol feeding occurred, NPC1L1 was internalized in *ApoE^shl^* but not in *ApoE^shl^* GM3S KO. Therefore, GM3s (or related gangliosides) are clearly essential for NPC1L1-dependent intestinal cholesterol absorption.

## 4. Human GM3S Deficiency

GM3S deficiency was first reported by A.H. Crosby’s group as an autosomal recessive infantile-onset epilepsy syndrome associated with developmental stagnation and blindness in Old Order Amish [51]. Biallelic pathogenic *ST3GAL5* variants led to disrupted synthesis of a- and b-series gangliosides, and consequently to severe infantile-onset neurological disorders characterized by progressive microcephaly, intellectual disability, choreoathetosis, blindness, deafness, intractable seizures, and/or pigment changes [51,52,53,54,55,56,57,58,59]. Reports of *ST3GAL5* mutations in patients with GM3S deficiency are summarized in Table 1. These patients appeared normal at birth, but all had psychomotor developmental delays, and most displayed consistent features of movement disorders and/or epilepsy. A “salt and pepper” syndrome sometimes seen in African American patients results from altered dermal pigmentation (hyper- or hypopigmented skin maculae) at various locations [54].

**Figure 2:**
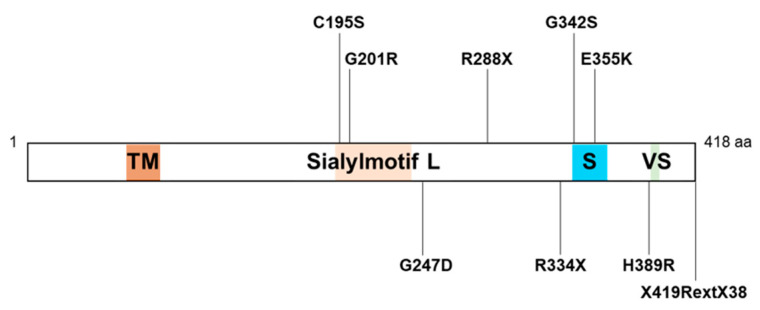
Sialyl motifs of ST3GAL5 and its variants detected in GM3S-deficiency patients (schematic) (see Section 4). Sialylmotifs L (large), S (small) and VS (very small) are highly conserved in mammalian sialyltransferases. X419RextX38 is a variant in the stop codon at the position 419, changing it to an R (arginine)-codon and adding a new amino acid extension with a new stop codon at position 38. TM, transmembrane domain.

The human GM3S protein consists of 418 amino acids and includes sequences for L- (large), S- (small), and VS- (very small) sialyl motifs (Figure 2), which are highly conserved in mammalian sialyltransferases [60]. A nonsense variant, R288X, detected in numerous Amish GM3S-deficiency patients, two French patients, and three Pakistani patients results in a truncated protein that lacks S and VS. motifs, and was therefore predicted to be nonfunctional [51,53,56]. Plasma GSL analysis of the Amish patients revealed a total absence of GM3 and its downstream derivatives, whereas increased levels were observed for LacCer (the direct substrate of GM3S), o-series gangliosides (e.g., GM1b), and globosides [57]. Several missense variants are located in L or S motifs: E355K (found in African American patients with salt and pepper syndrome), C195S and G201R (found as compound heterozygotes in Korean patients), and G342S (found in an Italian patient). In vitro GM3S assay for LacCer using homogenate of HEK293T cells transfected with mutated *ST3GAL5* constructs revealed an absence of enzymatic activity in these variants [58]. A recent study by C. Mignot’s group revealed four more novel variants: missense variants G247D and H389R, nonsense variant R344X, and stop-loss variant X419RextX38, which disrupts the stop codon and causes 37-codon extension of the reading frame [59]. Plasma GSL analysis of patients with these four variants showed significant reduction of GM3 levels; however, GM3 was still present (0.4–2.6 μmol/L in 7 patients, VS 11.5–14.4 μmol/L in 5 controls), as well as residual GM2—which was not observed in Amish patients with the R288X variant. The pathogenic mechanisms of these variants will be clarified by in vitro GM3S assay and analysis of intracellular localization and/or stability of ST3GAL5 protein. Two patients, homozygous for the above stop-loss variant, had the highest plasma GM3 levels (2.6 μmol/L) among the studied patients, and the least severe phenotype [59]. Plasma GM3 levels thus appear to be inversely related to severity of GM3S-deficiency disease.

In contrast to phenotypes of human GM3S-deficiency patients, GM3S KO mice do not display neuronal abnormalities, most likely because total brain ganglioside amount is roughly maintained through alternative expression of o-series gangliosides GM1b and GD1α, indicating that other gangliosides can compensate for lack of GM3 and its downstream biosynthetic derivatives (see Section 2). Whether such an alternative pathway is present in brains of human GM3S-deficiency patients is unknown. Substantial alternative o-series ganglioside expression was observed in fibroblasts from GM3S KO mice, but not in human GM3S-deficiency patients [61,62]. Causes of these differences in GM3S-deficient mice versus humans have not been experimentally studied, but two possible explanations can be considered: (i) differing expression levels of glycosyltransferases required for o-series ganglioside synthesis in Golgi; (ii) differing substrate specificity of mouse vs. human glycosyltransferases for o-series ganglioside precursors (LacCer, GA1, GA2). Expression of neutral GSLs (particularly Gb3 and Gb4) was elevated substantially in fibroblasts of human GM3S-deficiency patients, but only slightly in brains and fibroblasts of GM3S KO mice [53,54,61]. The mechanism underlying such difference in neutral GSL expression is unclear. Accumulated neutral GSLs may contribute to pathogenicity of GM3S deficiency in humans, possibly through alteration of receptor signaling and/or other cellular processes (e.g., mitochondrial function), as described for patient fibroblasts [53].

## 5. Conclusions and Future Perspectives

Physiological functions and pathological aspects of gangliosides revealed by studies on GM3S KO mice and human GM3S-deficiency patients are summarized in this review article. The cause of differential GSL expression patterns under GM3S KO conditions in humans versus mice is unknown but is presumably responsible for the striking differences in neurological phenotypes. Possible human-versus-mouse differences in expression levels or substrate specificity of enzymes involved in GSL synthesis are described in Section 4, and such differences may also exist for Golgi proteins other than enzymes. S. Parashuraman’s group demonstrated that Golgi protein GRASP55 binds to GlcCerS and LacCerS and prevents their retrograde transport, resulting in their accumulation in trans-Golgi [63]. Golgi protein GOLPH3 mediates retrograde transport of GSL-synthesis enzymes (LacCerS, GM3S, Gb3S), and thereby controls their sub-Golgi localization and lysosomal degradation rate [64]. If such fine glycosylation control processes are species-specific, they may account for differential human-vs.-mouse GSL expression patterns.

Nine distinct *ST3GAL5* variants have been detected in GM3S-deficiency patients. Four of them (G201R, R288X, R334X, E355K) are listed in the Genome Aggregation Database (gnomAD v2.1.1; https://gnomad.broadinstitute.org/, accessed on 26 May 2021) and have allele frequencies < 0.0001. In view of these very low frequencies, it is likely that many other deleterious *ST3GAL5* variants exist. Only four additional *ST3GAL5* variants in GM3S-deficiency patients have been reported since the first one. The 2022 study by C. Mignot’s group mentioned in Sec. 4 revealed four novel variants from 16 non-Amish patients (13 homozygotes, three compound heterozygotes) [59]. The authors expect that increasing numbers of novel variants will be detected in GM3S-deficiency patients, including ones currently undiagnosed. Serum GSL analysis is a noninvasive diagnostic technique for GM3S deficiency when GM3 and its downstream derivatives are undetectable or present in only trace amounts. The study by K. Strauss’s group demonstrated normal GM3 levels but elevated GlcCer, LacCer, and Gb4 levels in heterozygous carriers [57], indicating the usefulness of serum GSL analysis for carrier screening.

## Figures and Tables

**Table 1:** Clinical Features of patients with GM3 Synthase Deficiency.

Journal	Nat Genet/Am J Med Genet	Mol Metab Genet	Eur J Hum Genet	J Child Neurol		
Year	2004/2013	2019	2013	2018		
First Author	Simpson MA/ Wang H	Bowser LE	Fragaki K	Gordon-Lipkin E		
Descent	Old Order	Old Order	French	Pakistani		
	Amish	Amish				
Genotype	c.862C>T	c.862C>T	c.862C>T	c.862C>T		
	R288X	R288X	R288X	R288X		
	Homozygous	Homozygous	Homozygous	Homozygous		
Microcephaly	NR	50/50	NR	3/3		
Psychomotor delay	38/38	50/50	2/2	3/3		
Movement disorder	8/8	42/50	2/2	3/3		
Epilepsy	38/38	36/50	2/2	0/3		
Abnormal EEG	8/8	31/32	NR	3/3		
Sensorineural hearing impairment	NR	15/15	2/2	3/3		
Vision impairment	8/8	10/13	2/2	3/3		
Abnormal pigmentation	27/38	NR	NR	2/3		
**Journal**	**Hum Mol Genet**	**Am J Med Genet**	**Glycobiology**	**Genet Med**		
Year	2014	2016	2019		2022	
First Author	Boccuto L	Lee JS	Indellicato R		Heide S	
Descent	African-American	Korean	Italian	Reunion Island	Algerian	Italian
Genotype(s)	c.1063G>A	c.584G>C,	c.1024G>A	c.740G>A	c.1255T>C	c.1000C>T,
	E355K	c.601G>A	G342S	G247D	X419RextX38	c.1166A>G
	Homozygous	C195S, G201R	Homozygous	Homozygous	Homozygous	R334X, H389R
		Comp Het *				Comp Het
				c.740G>A,		
				c.1063G>A		c.1166A>G
				G247D, E355K		H389R
				Comp Het		Homozygous
						c.1024G>A,
						c.1166A>G
						G342S, H389R
						Comp Het
Microcephaly	4/4	1/2	1/1		9/16	
Psychomotor delay	4/4	2/2	1/1		16/16	
Movement disorder	3/3	2/2	1/1		14/14	
Epilepsy	1/4	0/2	1/1		12/16	
Abnormal EEG	NR	NR	1/1		NR	
Sensorineural hearing impairment	NR	NR	1/1		8/15	
Vision impairment	0/1	0/2	1/1		5/12	
Abnormal pigmentation	3/4	1/2	1/1		5/16	

* Comp Het, compound heterozygous.
